# Pseudo-Hypertrophic Paralysis

**Published:** 1888-05

**Authors:** 


### PSEUDO-HYPERTROPHIC PARALYSIS.

We have now to direct your attention to a little patient. She is five years of age, a bright, sprightly little girl, who has never walked. Notice the peculiar position of her body when she tries to stand or move forward. She tries to walk on her toes; she throws one foot in front of the other, and makes her progression on the toes. There is but little movement, and it is not made by bringing the foot down flat but by walking on the toes, throwing her head and chest away beyond the centre of gravity. She has some power of movement in the sitting posture, but it is much impaired. Touch-sense, temperature-sense, and pain-sense are normal.

There is one point to which I desire to call your attention in particular, and that is the condition of the thigh-muscles as compared with the calf-muscles. There is an enormous development of the calf-muscles. The tissues in the calf of the leg are hard and dense to the sense of touch. Notice the great overgrowth of tissue in the thigh. We have an overgrowth of the thigh-muscles, and just how much they are overgrown in comparison to what they ought to be as compared with the muscular structure generally, we cannot definitely determine. What calf-muscles she has are almost as hard as gristle. So far as the thigh-muscles are concerned they are hypertrophied, but it is not a hypertrophy with increased muscular power; there is loss of power; the tissues in her thighs and calves cannot possibly be proper muscular tissue. It does not perform the functions of muscular tissue, and it has a degree of density to the touch that does not belong to normal muscular tissue. The hypertrophy can not be due to overgrowth of muscular structure. Some change has taken place in the proper structure of the muscles; it is, in short, pseudo-hypertrophy, making this a case of pseudo-hypertrophic paralysis. The disease in this, as in the other case, is located in the anterior horns of the cord. It differs from the other case. The patient with poliomyelitis anterior acuta, who was just before you, up to the time of her fever and convulsions, had no impairment of power of the lower limbs. The acute process in her case came on suddenly. The trouble of this child has been going on since its early history. When the disease commenced we do not know. The early history is defective. We have here a chronic process. The trophic centres that are concerned in producing atrophy or impairment of function not only produce atrophy of muscular tissue, but a growth of adipose tissue sometimes instead of muscular tissue proper. Why this should be has never been satisfactorily explained.

This child was before the class last winter, and has made some improvement since that time. She is receiving now, and has been during the year, the constant galvanic current as strong as could be borne—a current of sufficient strength to produce muscular contractions—together with cod-liver oil, small doses of strychnia, and massage, and with this line of treatment judiciously carried out she has improved. If the muscles will respond to a current that the patient can tolerate, there is hope of restoration, and the treatment should be persisted in for months, and even years, if necessary. Just as long as the muscles will respond to a galvanic current there is hope of improving their nutrition and of restoring their power. Occasionally such cases recover.

